# Genetic Basis of Stress-Related Neuropsychiatric Disorders

**DOI:** 10.3390/genes15101274

**Published:** 2024-09-28

**Authors:** Dubravka Svob Strac

**Affiliations:** Rudjer Boskovic Institute, Division of Molecular Medicine, Bijenicka Cesta 54, 10000 Zagreb, Croatia; dsvob@irb.hr

Stress exposure is one of the major risk factors for the development of different psychiatric and neurodegenerative diseases, leading to various (mal)adaptive alternations in different parts of the brain, and affecting both brain structure and function [1,2]. However, other determinants (social, biological, genetic) could distinguish between persons who are at higher risk to develop neuropsychiatric disorders after exposure to stress from resilient individuals [3,4]. Hence, the identification of (epi)genetic underpinnings of resilience and vulnerability to stress is necessary in order to better understand the etiology of stress-related neuropsychiatric disorders, as well as to improve preventive and therapeutic strategies [5,6]. In the Special Issue “Genetic Basis of Stress-Related Neuropsychiatric Disorders”, we have collected a series of nine published papers, including five original research papers, three comprehensive literature reviews and one novel hypothesis. These articles cover different aspects of research in the field of genetics, pharmacogenetics, epigenetics, genomics, transcriptomics and proteomics, post-traumatic stress disorder (PTSD), anxiety and mood disorders, substance use disorders, schizophrenia and attention deficit hyperactivity disorder (ADHD).

Traumatic stress can affect various physiological mechanisms, which are linked with genetic variability. Genomics architecture plays an important role in the etiology of stress-related neuropsychiatric disorders, such as PTSD and behavioral disorders, including aggression. Research has suggested a link between aggressive behavior and PTSD, which may be influenced by the genes involved in the neurophysiological response to chronic stress and trauma. The review by Magwai and Xulu [7] summarized findings on genes or variants which have been investigated in both aggressive behavior and PTSD, as well as adverse childhood experiences. The authors focused on genes coding for monoamine oxidase A (*MAOA*), solute carrier family 6 member 4 (*SLC6A4*), brain-derived neurotrophic factor (*BDNF*), catechol-O-methyltransferase (*COMT*), dopamine receptor 2 and 4 (*DRD2* and *DRD4*) and FK506 binding protein 5 (*FKBP5*). The overview of these genes might add to better understanding of the genetic mechanisms contributing to aggression and PTSD.

Various genome-wide association studies (GWASs) have reported multiple genetic risk loci associated with PTSD; however, it is still not clear how they influence the risk of PTSD development. Progress in GWAS methodologies has further promoted the application of transcriptome-wide association studies (TWASs) and proteome-wide association studies (PWASs) in complex human diseases. Using a combination of GWAS, TWAS and PWAS data, Zhang et al. [8] focused on an analysis of genetic loci associated with PTSD, in order to elucidate the etiology of this complex trauma-related disorder. Through the FUSION pipeline, Zhang and colleagues first integrated two human brain proteome reference datasets (ROS/MAP and Banner) with the PTSD GWAS dataset to perform a PWAS analysis. Two transcriptome reference weights (Rnaseq and Splicing) were then applied to a TWAS analysis, and the PWAS and TWAS results were further studied using brain imaging analysis. Both the TWAS and PWAS analysis identified numerous candidate genes associated with PTSD, including *RIMS2*, *CHMP1A* and *SIRT5*, as well as *ADK* and *C3orf18*, respectively. The authors further compared the PWAS and TWAS results in different populations and detected overlapping genes—*MADD* in the total population and *GLO1* in female subjects—whereas brain imaging revealed different brain imaging phenotypes related to detected genes. These PTSD candidate genes, identified at both proteome and transcriptome levels, as well as the brain regions that are involved, may have contributed to the better comprehension of PTSD pathogenesis.

N-glycosylation is a highly conserved post-translational modification of proteins, which can change their biological role and might represent a connection between genetic background and environmental factors. Different pathophysiological states, including inflammation and PTSD, have been linked to modifications in N-glycome. Hepatocyte nuclear factor-1 (HNF1A) is a transcriptional regulator of various genes associated with inflammatory processes and regulates glycosylation of proteins in plasma. The study of Tudor and colleagues [9] evaluated the association of rs7953249 polymorphism in the *HNF1A* antisense RNA 1 gene (*HNF1A-AS1*), the rs735396 polymorphism in intron 9 of the *HNF1A* gene, and their haplotype block with the concentrations of plasma and immunoglobulin G (IgG) N-glycans in PTSD patients and control subjects. Additionally, methylation of four *HNF1A* CpG islands was investigated in PTSD and control subjects and further analyzed in relation to studied polymorphisms and concentrations of N-glycans in plasma and IgG. The authors observed significant association of *HNF1A* polymorphisms, as well as *HNF1A* gene methylation at the CpG3 site, with highly branched, galactosylated and sialyated plasma N-glycans, mostly in PTSD patients. Although the *HNF1A*-*AS1* rs7953249 polymorphism was associated with PTSD, none of the investigated polymorphisms were linked with methylation of the *HNF1A* gene. These findings suggest a possible regulatory role of the investigated *HNF1A* polymorphisms and their haplotypes in the regulation of the levels of complex plasma N-glycans previously associated with proinflammatory response. The involved mechanisms, possibly other than *HNF1A* methylation, could contribute to the clinical symptoms of PTSD and its comorbidities.

Both prenatal and postnatal environmental stressors can affect the gene networks and regulatory mechanisms in the hippocampus and play significant role in behavioral processes and neuropsychiatric diseases. The work of Rodriguez-Zas et al. [10] aimed to obtain a better insight into combined effects of viral maternal immune activation (MIA) and postnatal metabolic and inflammatory stressors on the hippocampus and potential sex differences. In a pig model of the viral MIA approach, the hippocampal transcriptome was determined on two-month-old female and male offspring using RNA sequencing. The effects of MIA were investigated in the groups with fasting metabolic challenge, viral mimetic inflammatory challenge and saline treatments as controls. Around 2600 genes were associated with single or combined effects of MIA, postnatal stress or sex, including messenger cyclic adenosine 3′,5′-monophosphate (cAMP), gastric inhibitory polypeptide receptor (GIPR), transcription factors C-terminal binding protein 2 (CTBP2), RE1 silencing transcription factor (REST), signal transducer and activator of transcription 1 (STAT1), and SUZ12 polycomb repressive complex 2 subunit. The results suggested the interaction of environmental challenges and their combined effects on transcriptome in the hippocampus. The presented findings could help in the identification of molecular targets, which could be used to diminish the negative consequences of pre-and post-natal stressors on hippocampal-associated physiology and behavior, and consequently the development of stress-related neuropsychiatric disorders.

Various studies have indicated that both prenatal and postnatal stresses influence a person’s risk for schizophrenia development and different neurodevelopmental, environmental and (epi)genetic factors (single or in combination) can contribute to schizophrenia pathogenesis. Epigenetic processes are involved in transcriptional activity, chromatin folding, cell division and apoptosis, as well as DNA damage and repair. Delphin et al.’s [11] revision examined some of the current literature on two main epigenetic regulation processes, DNA methylation and histone post-translational modification (PTM), in the brain and peripheral tissues of patients with schizophrenia and discussed the significance of epigenetic processes for schizophrenia diagnosis and treatment. The authors provided an overview of studies using various human tissues and techniques and highlighted the impact of epigenetic regulation in schizophrenia pathophysiology and pathogenesis, especially in brain development (pre- and post-natally). Moreover, since epigenetic modifications may act as predictors of treatment response and/or potential therapy targets, the authors underline the promising, but so far limited, clinical usage of pharmacoepigenetics in schizophrenia patients.

Schizophrenia is associated with substantially decreased life expectancy, with comorbid somatic diseases and, particularly, cardiovascular diseases being a major cause. Cardiac autonomic dysfunction (CADF) significantly increases cardiac mortality in patients with schizophrenia. Voltage-gated ion channels are widely distributed in the brain and heart, and therefore their aberrant function may link schizophrenia and CADF. Refisch and colleagues [12] addressed channel-encoding genes *CACNA1C* and *KCNH2* as promising candidate genes associated with both CADF and schizophrenia. The authors first searched the literature for *CACNA1C* and *KCNH2* polymorphisms, which demonstrated genome-wide significant association with schizophrenia, as well with CADF traits. The significantly associated polymorphisms observed, 5 *CACNA1C* and 9 *KCNH2*, were further studied in patients with schizophrenia and healthy controls and genotype-related impacts on heart rate (HR) dynamics and QT variability indices (QTvis) were investigated. An elevated QTvi was observed in schizophrenia patients, carriers of *CACNA1C* rs2283274 C and rs2239061 G risk alleles. Moreover, schizophrenia patients carrying *KCNH2* rs11763131 A, rs3807373 A, rs3800779 C, rs748693 G and 1036145 T alleles demonstrated a higher mean HR and QTvi. The study findings suggested a potential pleiotropic role for *CACNA1C* and *KCNH2* variations, linked with CADF in drug-free patients with schizophrenia, and suggested that CADF could represent an endophenotype of schizophrenia.

Pharmacotherapy of anxiety and depression has been marked by notable inter-individual variability in treatment response and occurrence of adverse effects. Pharmacogenetics represents a major part of personalized medicine, aiming to optimize treatment in accordance with a patient’s individual genetic signature by assessing genetic variations involved in pharmacokinetic or pharmacodynamic processes. The paper by Radosavljevic et al. [13] provided an overview of the most significant findings of pharmacogenetic and pharmacoepigenetic studies on antidepressants and anxiolytics. Pharmacogenetic studies investigating depression and anxiety have concentrated on genetic variants influencing metabolizing cytochrome P450 (CYP) and uridine 5′-diphospho-glucuronosyltransferase (UGT) enzymes; P-glycoprotein ATP-binding cassette (ABC) transporters; and monoamine and γ-aminobutyric acid (GABA) metabolic enzymes, transporters and receptors. Pharmacogenetic studies have suggested that more efficient and safer anxiolytic and antidepressant therapies might be achieved through genotype-guided decisions. Therefore, with accumulating evidence on the medical and economic benefits, improved guidelines, lower costs and shorter delivery times, pharmacogenetics may become a routine intervention in neuropsychiatric clinical practice. An emerging field of pharmacoepigenetics studies epigenetic mechanisms and their impact on individual responses to drugs. Further research on the interconnection between epigenetic modifications and drug responses is required in order to select more effective antidepressants and anxiolytics and minimize the likelihood of adverse reactions, consequently improving the quality of therapy.

Alcohol dependence (AD) is another stress-related neuropsychiatric disease with unclear etiology, which is affected by both genetic and environmental factors. Konjevod and colleagues [14] investigated the relationship between the functional polymorphism rs4290270 in the *TPH2* gene, which encodes tryptophan hydroxylase, the enzyme responsible for synthesis of serotonin in the brain, and both AD and personality traits, with a focus on Cloninger’s types of AD. Specifically, Cloninger type I AD is more determined by environmental factors, usually develops later in life (after age 25), and affects both genders equally, whereas type II AD has a strong genetic component, has earlier onset (prior age 25), and only influences men. Moreover, neurobiological deficits of patients with Cloninger type I AD are mostly related to the dopaminergic system, while type II AD patients have deficits primarily linked to serotonergic neurotransmission. The results demonstrated that the AA genotype and the A allele of the *TPH2* rs4290270 polymorphism were more common in AD patients in comparison to the control subjects. Additionally, a negative association was observed between the number of A alleles and Tridimensional Personality Questionnaire (TPQ) scores for harm avoidance in AD patients with type II—but not type I—AD, supporting various neurobiological mechanisms involved in the two types of AD. These results indicate the role of serotonergic genetic variations in AD pathogenesis, especially type II AD, and also suggest that in a subset of subjects, *TPH2* variation could potentially affect AD development by influencing the personality trait of harm avoidance. Therefore, this study highlights the significant influence of genetic variation on personality traits in individuals with different AD types.

Di Paola and al. [15] tested a working hypothesis which arises from methylation patterns in the 5′-UTR of the *DAT1* gene observed by analysis of data from children with attention deficit hyperactivity disorder (ADHD). The study considered relationships between CpGs pairs, of which one is located on the main gene strand and another on the complementary opposite strand (COS). In order to assess the probability that cytosines in such motifs might be all methylated (or not) simultaneously, the authors analyzed all possible combinations of probabilities (estimated by multiplying two raw values of methylation) in pairs of CpGs from either DNA strand. In addition, they suggested a crucial role for the “matrix-table”, calculating all correlations between any given pair and all other pairs of loci. Some pairs correlating with M6-M6COS had cytosines positioned to the reciprocal right (e.g., M3-M2COS and M6-M5COS), while other pairs had cytosines positioned to the reciprocal left (e.g., M2-M3COS and M5-M6COS). Significant pair-to-pair correlations were found between main strand and COS CpG pairs. Using graphic representations, the authors produced a hypothesis that the DNA folded to looping conformations, so the C1GG C2GG C3GG and C5G C6G motifs would become close enough to allow cytosines 1-2-3 to interact with cytosines 5-6 (on both strands). The findings suggested a sliding, with left- and right-ward oscillations of DNA strands. If the authors hypothesized correctly, the two alternative patterns of strand oscillations may either favor or contrast the continuation of DNA opening. Specifically, one of the two oscillations would tend to rotate the double helix to make it unwind and open, whereas the opposed oscillation would rotate the double helix to make it a supercoil, thus hindering further opening. While thorough empirical verification is needed, this hypothesis suggests that simultaneous methylation of main-strand and COS DNA (“methylation dynamics”) could be used as a potential biomarker in various medical conditions, including neuropsychiatric disorders.

In summary, the current Special Issue of *Genes*, “Genetic Basis of Stress-Related Neuropsychiatric Disorders”, presents recent data from research assessing genetic backgrounds, stress-related epigenetic modifications, and gene–stress interactions in different stress-related neuropsychiatric disorders, and it also provides a comprehensive overview of recent advances in the field, including genetic, pharmacogenetic and epigenetic approaches. Moreover, the papers collected in this volume underline the potential of innovative molecular approaches, such as GWAS, TWAS and PWAS, and various epigenetic analyses for the identification of (epi)genetic determinants of inter-individual variability in susceptibility to stress. The collected findings pave the way for further research in this field and could be utilized to obtain a better insight into the pathophysiology of neuropsychiatric disorders associated with stress, as well as to develop novel preventive and therapeutic strategies.

## Data Availability

Not applicable.
